# Emotional Meta-Memories: A Review

**DOI:** 10.3390/brainsci5040509

**Published:** 2015-11-09

**Authors:** Beth Fairfield, Nicola Mammarella, Rocco Palumbo, Alberto Di Domenico

**Affiliations:** 1 Department of Psychological Sciences, Health and Territory, University of Chieti, Via deiVestini 31, Chieti 66013, Italy; E-Mails: bfairfield@unich.it (B.F.); rocco.palumbo@unich.it (R.P.); alberto.didomenico@unich.it (A.D.D.); 2 Schepens Eye Research Institute, Harvard Medical School, 20 Stanford St., Boston, MA 02149, USA

**Keywords:** emotion, meta-memory, memory

## Abstract

Emotional meta-memory can be defined as the knowledge people have about the strategies and monitoring processes that they can use to remember their emotionally charged memories. Although meta-memory *per se* has been studied in many cognitive laboratories for many years, fewer studies have explicitly focused on meta-memory for emotionally charged or valenced information. In this brief review, we analyzed a series of behavioral and neuroimaging studies that used different meta-memory tasks with valenced information in order to foster new research in this direction, especially in terms of commonalities/peculiarities of the emotion and meta-memory interaction. In addition, results further support meta-cognitive models that take emotional factors into account when defining meta-memory *per se*.

## 1. Introduction

Meta-memory refers to an individual’s awareness of his/her memory processes and capacities, strategies for better memorization and the ability to monitor performance [1]. Meta-memory, in fact, enables an individual to reflect on and monitor his/her memory and the relationship between memory, knowledge about memory and the use of memory strategies has generated considerable theoretical and research interest, especially in the context of education and age-related differences [2].

Within meta-memory literature, most theorists (e.g., [3]) distinguish between declarative and procedural components of meta-memory. The declarative component corresponds to explicit knowledge about the contents and contexts of memory use and includes knowledge of content and capacity, knowledge of tasks and conditional knowledge about optimal memory performance. The procedural component, instead, includes control and monitoring subcomponents. The control subcomponent includes regulatory processes, such as planning, selection of relevant information, resource allocation decisions, selection of relevant strategies, and inferencing. The monitoring subcomponent includes a variety of self-assessment strategies, such as ease-of-learning judgments, judgments of learning prior to beginning a task, feeling-of-knowing judgments made during learning, and comprehension-monitoring judgments made during or after a task. In sum, the more an individual knows about memory, the better he/she can plan and monitor learning tasks.

Most importantly, although meta-memory awareness develops late and incrementally, it has an important impact on memory and cognitive performance. Moreover, it does not seem to be strongly linked to other cognitive factors, such as intelligence and memory capacity, but, rather, develops along with experience, feedback, and individual and group reflection.

In particular, different factors may account for individual differences in meta-memory: tasks demands and personality factors (such as state-based *vs.* trait-based conditions) are the most cited [4]. For example, many studies have shown that people with higher meta-memory skills are more likely to use memory strategies and show greater levels of recall than those with lower meta-memory [5,6]. Complex meta-cognitive models show that this may be due to the fact that meta-memory involves self-efficacy (that is, judgments and beliefs about one's performance capabilities with respect to the task at hand). However, a relevant source of individual variability in meta-memory may also be, generally speaking, emotion.

The aim of the current study is to review experimental evidence focusing on procedural components of meta-memory, that is, meta-memory tasks with a focus on emotionally charged information. Research findings are limited in this regard and do not allow us to draw clear conclusions. However, we believe that studying emotional meta-memory may help clarify the role of emotional factors in meta-cognition in general, and in the emotion-memory integration in particular, and better explain how people monitor emotional memory and whether or not they use distinct strategies to retrieve emotionally charged memories with respect to non-emotional ones. Furthermore, understanding emotional memory strategies may be ultimately relevant to situations where emotional memory is deficient or disrupted and may foster the development of specific emotional meta-memory training programs. Accordingly, we will focus on the possibility of identifying factors that may explain the peculiarity of emotional meta-memory with respect to meta-memory for non-emotional information and obtaining new insights related to emotion and meta-memory interaction.

### 1.1. Emotion and Meta-Memory

The affective domain is typically described according to two main emotional states: emotion and mood. Emotions refer to physical and psychological focused changes that influence our behavior, while mood is a more unfocused and diffused emotional state. There is general agreement among emotional theorists that emotional states can be organized along the dimensions of arousal and valence where arousal is objectively measurable as the activation of the sympathetic nervous system (although it can also be assessed subjectively) and valence is the subjective positive-to-negative evaluation of an experienced emotional state. In particular, valence may also be defined as the intrinsic attractiveness (positive valence) or averseness (negative valence) of an event, object, or situation. Here we will refer to this last definition (e.g., the dimension of stimulus valence) to explore the interaction between emotion and meta-memory and we will use the term “valenced information” throughout the paper to study meta-memory in relation to stimuli that are positively or negatively charged.

Generally speaking, with regards to valence (e.g., both as affective reaction and valenced information) involved in meta-memory, Efklides *et al.* [7] reviewed a series of studies that describe a broad interaction between valence and metacognition. In this case, positive emotions typically lower the person’s effort and increase interest and feelings of liking, thus supporting future engagement with the same or similar task and a consequent increase in cognitive performance. Again, positive mood also counteracts the effects of negative mood on feeling of difficulty and feeling of confidence. Differently, negative emotions inform the person that more effort is needed and that the task is more difficult than previously thought and thus may be considered as a signal to abandon the task. This data is in line with a recent study [8], which found that memory performance is sensitive to how participants interpret their study effort during the learning process. More recently, multidisciplinary findings also highlight an interaction between emotion and metacognition. For instance, Garfinkel *et al.* [9] show that the level of metacognition was modulated by stimulus presentation with respect to the phase of the heartbeat, further supporting the hypothesis that even automatic emotional reactions may affect metacognition. Another recent study [10], found that metacognition improved when participants revealed their confidence after making a decision under a worried mood. Generally speaking, these two findings indicate that the likelihood of remembering may increase as a result of an increased meta-memory emotion interaction.

Another significant area that may highlight the emotion modulation of meta-memory is aging and pathological populations such as, for example, schizophrenia patients. We believe that studying emotional meta-memory in these populations is an important research topic, given that differences in emotional processing exist. Altogether, the available reviewed data are in line with a trend to increasingly process positive and/or decreasingly process negative information, which is typically frontal lobe functioning based.

### 1.2. Meta-Memory Tasks

As previously said, a variety of procedural techniques have been developed and used to assess meta-memory. Here we focus on the most common (not exhaustive) list of tasks which include (a) Feeling-of-Knowing (FOK) judgments, in which participants estimate their likelihood of remembering material that they have previously studied and tried to recall; (b) Judgments-of-Learning (JOL) where participants estimate their likelihood of remembering items that have already studied; (c) Ease-of-Learning (EOL) where participants predict their memory based on a description of the task; and (d) Remember or Knowing (R/K), in which participants estimate how confident they are that an item was old because they can vividly recollect it or they just feel it was shown before.

Nelson and Narens [11] proposed a distinction between retrospective monitoring (e.g., a confidence judgment about a previous recall response) *vs.* prospective monitoring (e.g., a judgment about subsequent responding). For example, ease-of-learning judgments are predictions about what will be easy or difficult to learn, while R/K responses refer to previous responses. Although there may be some differences between these paradigms, as Dunlosky and Metcalfe [1] highlighted, they can be grouped together because they refer to any judgment that is about a memory, and, in our case, to any judgment about emotional memory. Therefore, although there are differences between tasks, all are supposed to tap online monitoring functions and thus invite participants to be more conscious of their level of performance during online and offline processing. Studying emotional meta-memories with a procedural (e.g., task-based) perspective is interesting because emotional events are remembered better than neutral events (the so-called emotional enhancement effect, [12]). In addition, emotional memories are believed to be more vivid compared to non-emotional ones [13]. Thus, better memory performance with emotional stimuli may also be due to better meta-memory for this type of stimuli.

To identify relevant studies, a comprehensive literature search in a variety of electronic databases was performed until July, 2015 (PubMed [14], PsychINFO [15], and Web of Science [16]). Entered search terms were “emotion,” “meta-memory,” “valence,” and “emotional memory.” In addition, reference lists from the retrieved articles were screened to identify additional papers. Articles were included for review if they met the following criteria: (1) The study used a meta-memory task that included emotional stimuli (2) Participants were younger and older adults and schizophrenia patients. The definition of emotion shows a large variation in the literature and may include a combination of different processes. Provided that valenced stimuli were involved, all studies were included. Altogether, we reviewed 13 studies.

## 2. Feeling-of-Knowing Judgments

Feeling-of-knowing (FOK) judgments ask for a prediction of the likelihood of subsequently recognizing currently unrecalled information. For example, a person may not be able to recall an item successfully, but can determine whether or not he or she will be able to recognize it from a list. In a typical paradigm, participants are asked to recall the target. Subsequently, if they are unable to retrieve it, they are asked to judge their feeling-of-knowing and then to recognize it. FOK can be given on either a Likert (*i.e.*, from 1 to 10) or a percentage scale (*i.e.*, 0–100). For example, responses on a Likert Scale to the FOK question “What are your chances of recognizing the correct target?” may be 1 = I definitely will NOT be able to recognize the target word/image to 10 = I definitely WILL be able to recognize the target word/image.

FOK depends on multiple factors, including the familiarity of the cue and accessibility to information about the encoding experience that may be diagnostic of target availability [1]. People often have better than chance accuracy in forecasting whether they will recognize unrecalled information [17,18,19,20]. Furthermore, subjective judgments are an accurate prediction of memory performance [21,22,23]. It is, of course, an intriguing thought that participants can accurately predict what they will or will not be able to recognize when their memory fails. One aspect is whether emotional valence may influence FOK.

The study by Thomas *et al.* [23] aimed to evaluate the influence of emotional contextual information according to the accessibility model. In general, this model posits FOK judgments are determined by the amount of partial contextual information accessed, regardless of its correctness [24]. Thomas *et al.* asked their participants to study paired associates that consisted in a neutral cue paired with a valenced target (positive or negative). Following study, participants took a cued recall test. If participants could not produce the target, they were asked to make a FOK judgment. After answering the FOK question, participants indicated the emotional connotation of the target by selecting one of three cues that appeared on the monitor: “good”, “bad” or “abstain”, in cases when they had no feeling about the target. In three experiments, the authors demonstrated that the emotional quality of contextual information modulated mean FOKs and FOK prediction accuracy. These findings suggested that the assessment of partial information, in this case valence, may have led to additional evaluation and search processes that triggered more accurate FOK and that FOKs might be sensitive to the type of materials used.

## 3. Judgment of Learning

In a typical judgment of learning (JOL) task, participants are asked to rate their confidence in the likelihood of remembering a studied item during a subsequent memory test. For example, after a word-pair presentation, participants are asked to indicate how likely they would be to recall the second word successfully if presented only with the first word on a subsequent test. They are asked to respond on a 5-point scale: 1 (definitely forget), 2 (probably forget), 3 (unsure), 4 (probably remember), 5 (definitely remember).

JOLs have been shown to depend on the ease with which the studied items are encoded or retrieved during learning. Consequently, emotional connotation may influence the way items are processed due to their high processing priority in attention and memory.

One of the first studies to use a JOL task to study the influence of emotion on meta-memory is the one by Zimmerman and Kelley [25]. The authors asked participants to make a JOL for positive, negative and neutral words. The authors found typical emotion enhancement effects but no specific valance effect indicating that JOL for emotional stimuli predicted better recall for emotional stimuli than neutral stimuli.

## 4. Ease of Learning

As far as we know, there are no studies that asked participants to judge the ease of learning during a memory task with emotional stimuli. From available literature on the ease of learning tasks, we assume that this type of task may also be sensitive to emotional variables. For example, Mazzoni *et al.* [26] examined the influence on memory monitoring and memory control of typicality and frequency of purchase of grocery items with an ease of learning task with food items as study items. The authors manipulated the food items to create three conditions: typically and frequently purchased, typically but not frequently purchased and not typically and not frequently purchased. Results highlighted how low frequency items were more difficult to remember and, in particular, how judgments about the ease of remembering each single item was influenced by the pre-acquisition characteristics of the items.

Confidence was measured by asking participants to answer on a 6-point scale (from 1 = 100% sure that the item was old to 6 = 100% sure that the item is new). Usually, food items are thought to be valenced items and self-relevant based. It is therefore possible that emotional connotation, in addition to frequency of purchase, influenced findings as well.

Kelemen *et al.* [4] also conducted a study using an ease of learning task. In this study, participants completed each task twice, with a one-week interval between sessions. This methodology allowed the authors to examine the stability of individual differences over time within a given task. In particular, a sequence of word pairs was presented one at a time and participants provided a (self-paced) judgment as to how difficult each pair would be to learn. Following all EOL ratings, participants studied the paired associates for 6 s each. After studying all paired associates, participants completed a 3 min distractor task. A self-paced cued-recall test was then administered. This study showed that while memory and confidence levels for items were stable, metacognitive accuracy was not. The authors claimed that metacognitive accuracy can change over time intervals based on individual affective characteristics of participants (mood, depression). However, mood and emotional factors were not directly examined in this study. In addition, these data are in line with the assumption of a previous study [8] such that metacognitive judgments depend on both data-driven and goal-driven effort. In this case, data-driven effort is the amount of effort required by the task in a bottom-up fashion, while goal-driven effort refers to the amount of effort invested in a task in a top-down fashion. Emotion in general, and valence in particular, may exert an influence at both levels of effort.

## 5. R/K Responses

Another method that has been used to study states of awareness accompanying memory for emotional events is what has become famous as the remember/know (R/K) paradigm. In this procedure, when an item is recognized as old on a memory test, participants are asked to decide whether they remember that item (*i.e.*, they can recollect specific details about that item and its memory is vivid) or if they just know that it was seen previously (*i.e.*, the item seems only familiar).

Ochsner’s study [27] is one of the first to have used the remember/know paradigm to investigate the effect of emotion during recognition. In particular, across three experiments, the author asked participants to study a series of emotional pictures that differed in terms of valence and arousal. In a subsequent session, participants were asked to discriminate between old and new items. If the item was judged as old, they were asked to respond R (remember) or K (know). This study showed that negative and high-arousing stimuli received a larger number of R responses, while positive stimuli tended to receive a larger number of K responses. The authors concluded that when participants are invited to express their sense of recognition using the R/K paradigm for emotional stimuli, negative and high-arousing stimuli are qualitatively re-experienced in memory in a different manner compared to positive and low-arousing stimuli and neutral ones.

Sharot *et al.* [28] asked participants to study a series of emotional and neutral pictures first and then subsequently scanned them via fMRI (functional magnetic resonance imaging) while indicating whether they remembered the item or just knew it. The main findings were that “remember” responses for emotional and neutral items are associated with distinct neural activity. The amygdala and parahippocampus, two medial temporal lobe structures, are linked to two independent memory systems. Each structure has unique characteristic functions but in emotional situations, these two systems interact in subtle but important ways. Specifically, the amygdala can modulate both the encoding and the storage of hippocampal-dependent memories while the hippocampal complex, by forming episodic representations of the emotional significance and interpretation of events, can influence the amygdala response when emotional stimuli are encountered. In particular, enhanced activity in the amygdala was observed during “remembering” of emotional items, while parahippocampal regions were involved during “remembering” of neutral items.

More recently, Mickley and Kensinger [29] applied the R/K paradigm to emotional words and pictures in a neuroimaging study in order to examine the impact of the valence of emotional information on the encoding processes corresponding with later remembering and later knowing. Their participants underwent a surprise recognition test in which they were shown a series of words and pictures on a computer screen and asked to indicate whether each item was one that they had been previously seen. If participants indicated that an item had been seen, they were then asked whether the item was vividly “remembered” or just “known” to be familiar. Their results add support to the hypothesis that the processing of positive information is associated with a higher number of know responses that an item is familiar, but not to remember the episodic details of its presentation. In summary, the valence of emotional information has an impact on the later remembering *vs.* knowing responses.

## 6. Aging, Schizophrenia and Emotional Meta-Memory: Is There a Special Relationship?

Many studies have shown that memory for emotional information is maintained better than memory for neutral information in aging [30,31,32,33,34,35,36,37,38]. For example, older adults remember their own internal emotional reactions more vividly than younger adults do [39]. In addition, older adults can remember source information (which requires monitoring and evaluation of encoded events) as well as younger adults if the source is identified via its emotional rather than neutral components [40]. This data suggests that older adults are more likely to adopt an emotional focus when encoding and retrieving memories events. Consequently, one may expect emotional meta-memory to be preserved in aging despite their diminished meta-memory accuracy [41]. In fact, neuroimaging studies suggest that meta-memory relies on frontal and medial temporal lobes, which show a reduced activation in both healthy aging and Alzheimer’s disease ([42] for a review). Noteworthy, however, meta-memory studies with healthy aging are rather mixed, with some studies reporting significant declines, and others suggesting that meta-memory may be preserved in aging. Although, these differences across studies may be due to the different methodologies used [43], none, as far as we know, has taken into consideration that variations could be due to different degrees of emotional meta-memory factors involvement. This claim is in line with the study by Sacher *et al.* [44], which suggested that meta-memory deficits in older adults may be due to shallow encoding processes, leading to difficulty retrieving target-related contextual details.

A study by Kapucu *et al.* [45] using R/K responses found that both younger and older adults showed a comparable number of old and “remember” responses on emotional stimuli, although differently biased (with a negativity bias in younger adults since younger adults showed better performance for negative items while and a general emotional bias in aging since older adults for both positive and negative items with respect to neutral ones).

Another study by Comblain *et al.* [46], investigated age-related differences in recognition memory for emotional and neutral pictures. At study, younger and older adults rated pictures according to valence, arousal and visual complexity. Following a two-week interval, participants recognized the pictures and the states of awareness associated with memory were assessed with the R/K paradigm. Results showed that "remember" responses in older adults were more often based on a recollection of emotional reactions rather than on the stimulus emotional connotation *per se*.

In another interesting study [26], the authors investigated how aging may affect emotional meta-memory with a JOL task. Again, an emotional enhancement effect was found, but this time a valence effect also occurred, as older adults’ JOLs were higher for negative than neutral words compared to younger adults. This finding highlighted our processing priorities may change as we age and influence both JOLs and memory performance.

As for aging, another interesting pattern of results may be found in schizophrenia literature. Herbener [47] summarized literature regarding the interaction between memory and emotion and found mixed results in terms of an emotional enhancement effect. Generally speaking, with regards to meta-memory tasks, schizophrenia patients seem to show a slight advantage for emotional information. For example, a study by Danion *et al.* [48] tested schizophrenia patients and healthy controls with an R/K procedure with emotional words and found that patients recollected emotional words more frequently than neutral words. On the contrary, “know” responses were not influenced by emotional connotation. Differently, another study [49] using an R/K procedure with emotional pictures did not highlight advantages in recollection for emotional items in the patients group. More recently, Peeters *et al.* [50] presented a series of video sequences that differed in emotionality to a group of schizophrenia patients and a group of healthy controls. After each video, participants made all/new discriminations with confidence ratings. The authors found a reduced recognition in the patient group for negative and neutral items. Most interestingly, meta-memory was not influenced by the emotional connotation of video. Altogether, these studies on schizophrenia patients suggest that these patients perform worse in accuracy and show higher confidence responses for errors with respect to healthy controls. However, we believe that a better understanding of emotional meta-memory in schizophrenia should take into consideration different meta-memory tasks and not only R/K procedures. In addition, the well-known deficit of patients in executive functions [51] may be called on to explain emotion processing and different emotional goals that lead to differences in emotional meta-memory (e.g., whether patients show better meta-memory for emotional *vs.* neutral information).

## 7. Conclusions

The reviewed data about meta-memory and emotion interactions lead to several considerations. First, the way emotion is studied makes a difference. For example, the distinction between mood and emotion is crucial to better understanding relevant implications for meta-memory. No meta-memory studies, as far as we know, have directly manipulated emotional congruence in term of mood and stimulus valence.

Second, a well-established line of research on positive information [45,46] has shown that negative and positive emotions differentially affect cognition. Meta-memory for differently valenced information is sensitive to these aspects as well.

Third, differences in the type of affective material are relevant as well. Pictures, in fact, may create richer memory traces and generate larger emotional enhancement effects and differentially affect meta-memory tasks.

A final thought must be directed towards a motivational explanation of meta-memory data since motivation has been shown to be crucial to understanding meta-memory. In fact, lack of motivation is considered as one of the main characteristic of meta-memory disruption. Given that motivation towards meaningful goals requires the recruitment of cognitive resources, results may depend on the lack of attentional and memory processes motivated to emotion processing.

To conclude, studying emotional processing in meta-memory is relevant since there may be emotional gains for meta-memory as well. Undeniably, people can use emotions to foster their meta-memory and this may have relevant implications in different contexts. Emotional meta-memory integrity, then, may help increase general activity levels, motivation for undertaking new activities, and sense of competence, leading to better outcomes. We hope that the evidence briefly reviewed in this study fosters both emotion research in the field of meta-memory and give new insights into meta-memory models.
